# The Diagnostic Role of the Platelet-to-Lymphocyte Ratio in Ovarian Cancer: A Systematic Review and Meta-Analysis

**DOI:** 10.3390/ijms26051841

**Published:** 2025-02-21

**Authors:** Magdalena Bizon, Maciej Olszewski, Boguslawa Krason, Elzbieta Kochanowicz, Kamil Safiejko, Anna Borowka, Joanna Sekita-Krzak, Michal Pruc, Anna Drozd, Stepan Feduniw, Basar Cander, Lukasz Szarpak

**Affiliations:** 1 Department of Clinical Research and Development, LUXMED Group, 02-678 Warsaw, Poland; 2 LUX MED Oncology Hospital, 01-748 Warsaw, Poland; 3 Department of Gynecology and Obstetrics with Gynecology Oncology Subdivision, MEGREZ Hospital, 43-100 Tychy, Poland; 4 Institute of Biological Science, Collegium Medicum, The John Paul II Catholic University of Lublin, 20-708 Lublin, Poland; 5 Colorectal Cancer Unit, Maria Sklodowska-Curie Bialystok Oncology Center, 15-027 Bialystok, Poland; 6 Collegium Medicum, The John Paul II Catholic University of Lublin, 20-708 Lublin, Poland; 7 Institute of Medical Science, Collegium Medicum, The John Paul II Catholic University of Lublin, 20-708 Lublin, Poland; 8 Department of Public Health, International European University, 12331 Kyiv, Ukraine; 9 Department of Oncology, European Health Center Otwock, 05-400 Otwock, Poland; 10 Department of Gynecology, University Hospital Zurich, 8091 Zurich, Switzerland; 11 Department of Emergency Medicine, Bezmialem Vakif University, Fatih, 34093 Istanbul, Turkey

**Keywords:** ovarian cancer, platelet-to-lymphocyte ratio, biomarker, meta-analysis, systematic review

## Abstract

Ovarian cancer is among the most lethal gynecologic malignancies, often diagnosed at advanced stages due to a lack of effective screening tools. Recent studies suggest that the platelet-to-lymphocyte ratio (PLR), an indicator of systemic inflammation, may serve as a potential biomarker for diagnosing and staging ovarian cancer. We conducted a systematic review and meta-analysis, adhering to PRISMA guidelines. We searched the PubMed/Medline, Scopus, Web of Science, and EMBASE databases. We pooled data using a random-effects model to assess the sensitivity, specificity, and diagnostic performance of PLR in ovarian cancer. The meta-analysis of 22 studies comprising 5740 participants showed significantly elevated platelet-to-lymphocyte ratio (PLR) values in ovarian cancer patients compared to healthy controls, with a mean difference of 46.84 (*p* < 0.001). Additionally, PLR demonstrated utility in distinguishing benign from malignant lesions and early-stage from advanced-stage ovarian cancer. While PLR shows potential as a cost-effective and accessible biomarker for ovarian cancer diagnosis and staging, its diagnostic accuracy remains moderate. Therefore, combining PLR with other diagnostic tools enhances clinical decision-making.

## 1. Introduction

Ovarian cancer (OC) is one of the most lethal gynecologic malignancies globally, with over 300,000 new cases and nearly 200,000 fatalities each year [1]. The GLOBOCAN 2020 database from the International Agency for Research on Cancer (IARC) reveals that OC is the eighth most prevalent cancer among women and the seventh most significant cause of cancer-related death in this group. In 2020, the database forecasted a global age-standardized incidence rate of OC at 6.6 per 100,000 and a global age-standardized mortality rate of 4.2 per 100,000. GLOBOCAN 2020 estimations project that by 2040, the global incidence of OC in women will rise by approximately 42%, resulting in an extra 445,721 cases. The annual mortality rate from OC is anticipated to rise significantly, increasing by nearly 50% relative to 2020, with an estimated total of 313,617 deaths [2].

The disease’s high mortality rate is mainly due to its asymptomatic progression in initial stages and the absence of effective screening techniques, leading to most diagnoses being made at advanced stages. Survival outcomes vary significantly based on the stage at diagnosis: the 5-year survival rate exceeds 90% for patients diagnosed with stage I disease but falls below 30% for those diagnosed at stage III or IV [3]. The etiology of OC is multifactorial, encompassing both genetic predispositions and environmental influences. Germline mutations in the BRCA1 and BRCA2 genes are recognized as significant factors, with carriers facing lifetime risks of 40–50% and 10–20%, respectively [4]. Researchers have identified more genetic abnormalities strongly linked to ovarian cancer, including mutations in TP53, PIK3CA, and KRAS, with varying frequencies across various subtypes of the disease. The manifestation of P53 mutation is the most commonly seen mutation in high-grade serous ovarian carcinoma. The mutation rate of P53 escalates to 54.5% in high-grade serous ovarian carcinoma. Conversely, most hereditary ovarian cancer cases are attributed to BRCA1/2 genes. The BRCA mutation prevalence escalates to 40% in recurrent high-grade serous ovarian carcinoma. PIK3CA mutations are frequently seen in ovarian clear-cell carcinoma and endometrioid ovarian cancer associated with endometriosis. The existence of the KRAS mutation is a crucial element in the progression of low-grade serous ovarian cancer and mucinous ovarian cancer. The potential associations between these mutations and ovarian cancer encompass several processes, including the disruption of tumor suppressor genes, deficiencies in DNA repair genes, modifications in apoptosis, the activation of oncogenes, and epigenetic silencing [5].

Environmental factors, such as nulliparity, late menopause, and endometriosis, also elevate OC risk, consistent with the ‘incessant ovulation’ hypothesis [6]. Recent evidence highlights a significant correlation between endometriosis and an elevated risk of ovarian cancer. Women with endometriosis have a 4.2-fold heightened chance of developing ovarian cancer relative to those without the illness. Women with ovarian endometriomas and/or deep infiltrating endometriosis demonstrate a 9.7-fold elevated risk relative to those without endometriosis. The associations between endometriosis subtypes and ovarian cancer histotypes are much more pronounced for type I (endometrioid, clear cell, mucinous, and low-grade serous) than for type II (high-grade serous) OC. This demographic may benefit from counseling about ovarian cancer risk and prevention and might provide a substantial cohort for targeted screening and preventative research [7]. Endometriosis has been identified as a significant risk factor for ovarian cancer, particularly for type I tumors, such as endometrioid and clear-cell carcinomas. Molecular mechanisms linking endometriosis to ovarian carcinogenesis include chronic inflammation; oxidative stress; and the activation of oncogenic pathways, such as PI3K/AKT and ARID1A mutations. These pathways may differ from those driving sporadic ovarian cancer, which often involves BRCA mutations and TP53 alterations. Additionally, the presence of endometriotic lesions creates a microenvironment rich in cytokines and growth factors, promoting cellular proliferation and genomic instability, which may further contribute to malignant transformation.

Notwithstanding improvements in surgical methods and chemotherapy, the prognosis for advanced-stage ovarian cancer remains unfavorable, underscoring the critical necessity for innovative diagnostic and therapeutic approaches. In recent decades, substantial advancements have been achieved in the exploration and identification of novel, sensitive, specific, and accurate OC biomarkers, significantly enhancing personalized medicine and improving cancer patient outcomes, particularly through the development of molecular biology technologies for OC biomarker detection. Recent advancements in biomarker identification, such as CA125, HE4, and circulating tumor DNA (ctDNA), offer potential for earlier detection [8,9]. While CA125 remains the most widely used biomarker in clinical practice for ovarian cancer detection, its sensitivity and specificity are limited, particularly in early-stage disease. HE4 has shown promise as a complementary marker, especially in combination with CA125, but its use is not yet universally adopted. Circulating tumor DNA (ctDNA) is still primarily in the experimental and validation stages, offering potential for early detection but requiring further research. In comparison, PLR, as an inflammatory marker, has demonstrated moderate diagnostic accuracy (AUC = 0.664) and may serve as a cost-effective adjunct to traditional biomarkers, particularly in resource-limited settings. Molecular profiling of tumors has enabled the creation of targeted therapies, including Poly (ADP-ribose) polymerase (PARP) inhibitors for BRCA-mutated cancers, which enhance survival rates. Targeted therapy has revolutionized oncological therapies for cancer, demonstrating significant effectiveness and a more acceptable side-effect profile relative to conventional chemotherapy. PARP inhibitors have shown success in the treatment of BRCA1/BRCA2 mutant malignancies, particularly in breast and ovarian cancers. Cancer cells have an inherent capacity to mutate, hence developing resistance to treatment [10]. In light of these insights, initiatives to mitigate the global burden of OC must emphasize the creation of dependable, accessible, and economical diagnostic instruments. Simultaneously, public health initiatives focused on enhancing awareness and executing screening strategies in high-risk populations are essential. Ongoing investment in research examining the molecular pathogenesis of ovarian cancer is essential for attaining substantial advancements in early detection and patient outcomes.

Among the novel biomarkers, indicators of systemic inflammatory response, such as the platelet-to-lymphocyte ratio (PLR), have attracted interest [11]. PLR, calculated as the absolute platelet count divided by the lymphocyte count, reflects systemic inflammation and immune dysregulation. In ovarian cancer, an elevated PLR is thought to arise from tumor-induced thrombocytosis and lymphopenia, which correlate with immunosuppressive microenvironments and metastatic spread [12].

**Figure 1:**
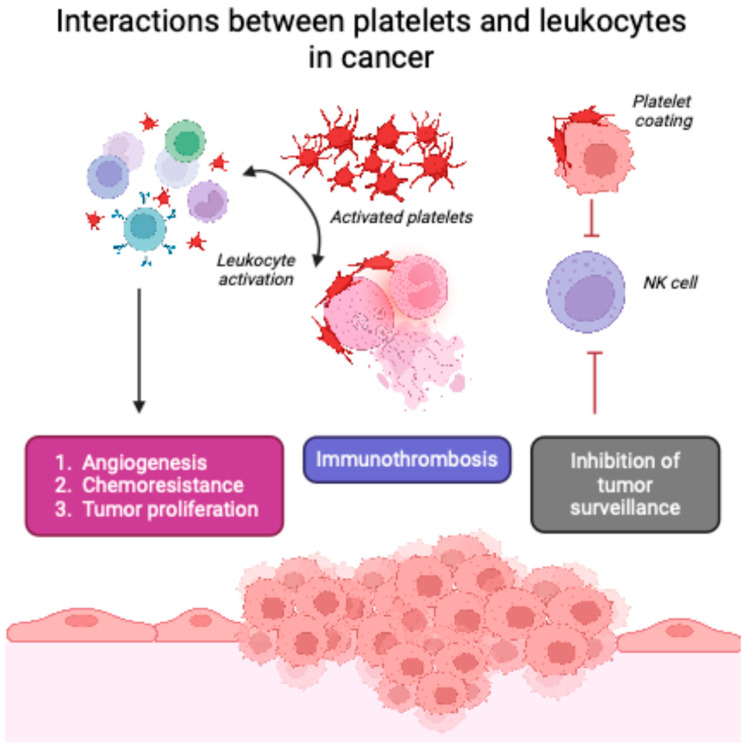
Platelet–leukocyte interplay in cancer development and progression.

The interplay between platelets and lymphocytes significantly influences cancer development. Platelets can form aggregates with lymphocytes, particularly neutrophils and monocytes, through adhesion molecules such as P-selectin. This interaction promotes the release of pro-inflammatory cytokines and growth factors, creating a tumor-supportive microenvironment. Additionally, platelet–lymphocyte aggregates can facilitate the formation of neutrophil extracellular traps (NETs), which have been implicated in capturing circulating tumor cells (CTCs) and aiding their extravasation into distant tissues, thereby promoting metastasis (Figure 1). Activated platelets may directly interact with circulating tumor cells, facilitating immune evasion and metastasis. While this mechanism is biologically plausible, our analysis focused on PLR as a systemic marker rather than localized platelet–tumor interactions.

Chronic inflammation is crucial in the onset, advancement, and spread of cancer, acting as a significant factor in carcinogenesis and the malignant transformation of cells. Ovarian cancer has been examined with respect to systemic inflammatory indicators, notably the platelet-to-lymphocyte ratio (PLR), indicative of a pro-inflammatory condition. Increased PLR levels have been linked to worse prognosis; advanced disease stages; and heightened metastatic potential in several malignancies, including ovarian cancer [13,14,15]. In contrast to conventional tumor markers like CA125, which are fundamental to the diagnosis and surveillance of ovarian cancer, PLR offers several benefits. It is economical, non-invasive, and easily accessible via standard complete blood count (CBC) testing. Furthermore, PLR provides significant insights into the inflammatory and immunological dynamics of the tumor microenvironment, possibly enhancing current diagnostic and prognostic instruments [16,17]. Recent studies have examined the application of PLR in several therapeutic scenarios, including in differentiating benign from malignant ovarian tumors, assisting in cancer staging, and forecasting disease recurrence. Nevertheless, the results concerning the diagnostic and prognostic relevance of PLR in ovarian cancer are conflicting. Some studies emphasize its promise as a dependable and pragmatic biomarker, while others stress its limits, noting overlapping results with benign circumstances and inconsistency in predictive efficacy. These discrepancies highlight the need for a thorough meta-analysis to consolidate the existing evidence, assess the diagnostic precision of PLR, and discern potential variables affecting its efficacy. This investigation might elucidate the potential integration of PLR as a reliable supplementary marker in the therapy of ovarian cancer within clinical practice.

The aim of this systematic review and meta-analysis is to determine the overall sensitivity and specificity of the platelet-to-lymphocyte ratio in ovarian cancer and its potential as a diagnostic tool.

## 2. Materials and Methods

This review was performed according to the Preferred Reporting Items for Systematic Reviews and Meta-Analyses (PRISMA) statement [18] (Table S1). The protocol was registered in PROSPERO (CRD42024605168). The review is based on aggregated data that have already been published in the international literature. Therefore, we waived patient consent and institutional review board approval.

### 2.1. Search Strategy

Two independent researchers conducted a comprehensive search across various databases, including PubMed/Medline, Scopus, Web of Science, and EMBASE, for papers written in English and published up to November 2024. The primary objective was to identify studies reporting the influence of the platelet-to-lymphocyte ratio (PLR) in ovarian cancer in women. The search strategy incorporated the following terms: “blood platelets and lymphocytes” OR “PLR” OR “platelet-lymphocyte ratio” AND “ovarian neoplasm” OR “ovarian neoplasms” OR “ovary neoplasm” OR “ovary neoplasms” OR “ovary cancer” OR “ovary cancers” OR “ovarian cancer” OR “ovarian cancers” OR “cancer of ovary” OR “cancer of the ovary”. This was supplemented by a retrospective reference list of the included literature, relevant published systematic reviews, and meta-analyses to include, as fully as possible, the literature that meets the inclusion criteria. While several meta-analyses [19,20] have explored the role of PLR in various cancers, including ovarian cancer, this study distinguishes itself by focusing specifically on the diagnostic accuracy of PLR in differentiating ovarian cancer from benign conditions and healthy controls. Unlike previous reviews, our analysis incorporates a broader range of studies and employs rigorous statistical methods to account for heterogeneity, providing a more comprehensive evaluation of PLR’s diagnostic potential.

### 2.2. Inclusion Criteria

Inclusion criteria: (1) original research; (2) studies using PLR as a biomarker for diagnosing ovarian cancer; and (3) studies published in English.

Exclusion criteria: (1) studies with only experimental groups; (2) studies on cell experiments and animal experiments; (3) studies with invalid data; (4) studies that were letters, conference abstracts, reviews, editorials, and master’s theses; and (5) repeated research.

### 2.3. Literature Screening and Data Extraction

**Figure 2:**
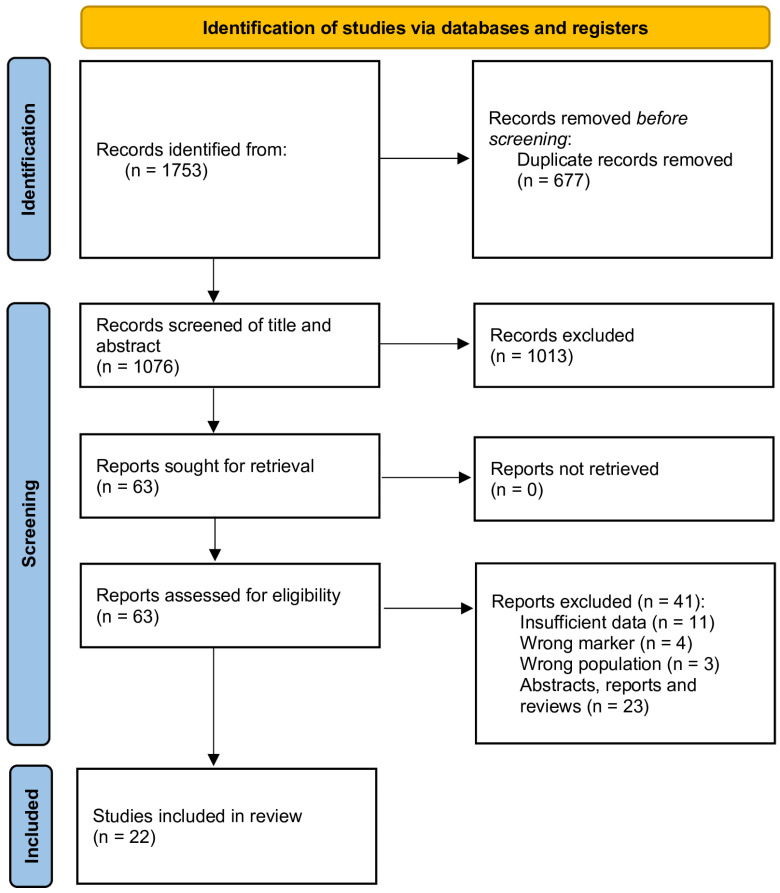
PRISMA flow chart.

We used Endnote to manage the literature and eliminate duplicate studies. We sequentially eliminated irrelevant studies by reading their titles and abstracts. Full-text downloading was conducted for studies that remained. Figure 2 illustrates the PRISMA flow diagram for research selection.

Two researchers (M.B. and M.P.) independently conducted the initial screening and data extraction according to the inclusion and exclusion criteria; a third researcher (L.S.) was consulted for assistance in divergent studies to obtain the target studies. We extracted data from the included studies using Excel 2021. The information extracted comprised (1) study characteristics (i.e., title, first author, country, study design, year of publication); (2) patient characteristics (i.e., sex, age); (3) PLR values among ovarian cancer and comparator groups; (4) true-positive (TP), false-positive (FP), false-negative (FN), and true-negative (TN), which should have been reported or could be calculated by using the following diagnostic properties: sensitivity (SEN), specificity (SPE), positive predictive value (PPV), negative predictive value (NPV), the positive likelihood ratio (PLR), the negative likelihood ratio (NLR), and the area under the receiver operating characteristic curve (ROC-AUC).

### 2.4. Quality Assessment

Two review authors (M.B. and M.O.) independently assessed the methodological quality of each study according to the Newcastle–Ottawa criteria [20]. We assigned a maximum of 9 points to each study, which included 4 points for selection, 2 points for comparability, and 3 points for outcome assessment. We classified studies with scores ranging from 0 to 3, from 4 to 6, and from 7 to 9 as low, moderate, and high quality, respectively. Discussions with the senior author (L.S.) resolved any potential disagreements.

### 2.5. Statistical Analysis

We performed data synthesis and statistical analyses of this research using REVMAN (Version 5.4.1). The threshold for statistical significance was established at a two-tailed α level of <0.05. The mean and standard deviation (SD) were used for calculations of the mean difference between the baseline and study follow-up. We used the Hozo formula for pooled statistics to convert studies reporting medians (interquartile ranges) to means (SDs) [21]. Studies reporting the mean with SE were converted to the mean SD using the following formula: SD = SE × $\sqrt{n}$ (where $\sqrt{n}$ is the square root of the sample size). Similarly, studies reporting the mean 95% CI were converted to the mean SD using the following formula: SD = (upper limit of CI − lower limit of CI)/(2 × 1.96), where CI is the 95% CI. The pooled mean differences (MDs) with 95% confidence intervals (CIs) were calculated to evaluate the relationship between the level of PLR and the diagnosis of ovarian cancer patients. A random effects model was used for the statistical model, as it allows for heterogeneity of results across studies [22].

The Chi-square test and I^2^ test were applied to evaluate the heterogeneity across studies. I^2^ values below 25% indicated low heterogeneity in the included studies, while 50% and 75% were considered moderate and high heterogeneity, respectively [23]. We conducted sensitivity analyses to assess the robustness of the results. We serially excluded each included study in the analysis to determine whether any investigation had a disproportionate influence on the meta-analysis results.

The diagnostic meta-analysis encompassed pooled sensitivity, specificity, the positive likelihood ratio (PLR), the negative likelihood ratio (NLR), the diagnostic odds ratio (DOR), and the construction of a summary ROC (sROC) curve. Variations in diagnostic thresholds across individual tests can induce a threshold effect, a significant contributor to heterogeneity in diagnostic meta-analyses. We computed the Spearman correlation coefficient between sensitivity and specificity to investigate whether a strong negative association exists, suggesting the presence of a threshold effect. The accuracy was delineated based on AUC: B 0.5, which indicates low accuracy; B 0.7, which signifies moderate accuracy; B 0.9, which represents good accuracy; and 1, which denotes perfect accuracy [24]. If the number of studies was not sufficient to calculate the pooled AUC, we could use the DOR. An elevated DOR also signified superior diagnostic efficacy.

## 3. Results

### 3.1. Study Selection

We identified 1076 publications (following the elimination of duplicates), of which 1013 were removed after screening titles and abstracts, resulting in 63 papers for comprehensive assessment. Two supplementary publications were discovered by manual examination of the reference lists. Subsequent to reviewing the complete text, an additional 41 studies were removed for the reasons stated in Figure 2. Ultimately, this evaluation encompassed 22 research studies, including a total of 5740 participants [25,26,27,28,29,30,31,32,33,34,35,36,37,38,39,40,41,42,43,44,45,46]. The high exclusion rate (1013 → 22 studies) primarily reflects stringent inclusion criteria, such as the requirement for studies to report PLR values in both cases and controls, and the exclusion of non-English articles, case reports, and studies lacking key outcomes (e.g., sensitivity/specificity). Many excluded works focused on other inflammatory markers (e.g., NLR) or did not stratify data by ovarian cancer subtypes, limiting their relevance to our meta-analysis.

### 3.2. Study Characteristics

The included studies were published between 2015 and 2024. The sample sizes for each study ranged from 36 to 665, and a total of 5740 patients were included in this study. Nine studies were conducted in China, 9 in Turkey, 3 in Korea, and one in Egypt. The evaluation quality results and summary are shown in Table S2. All of the studies were moderate/high quality.

### 3.3. Pooled Analysis of PLR Values

**Figure 3:**
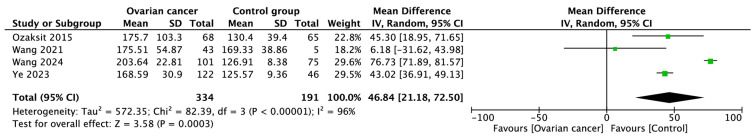
Forest plot demonstrating PLR values among patients with ovarian cancer and healthy groups [36,40,41,42].

Four studies reported PLR values in ovarian cancer and control groups. The pooled analysis of PLR revealed a mean value of 181.53 ± 57.02 for ovarian cancer patients and 128.89 ± 25.51 for the control group (mean difference [MD] = 46.84; 95% confidence interval [CI]: 21.18–72.50; *p* < 0.001; Figure 3).

**Figure 4:**
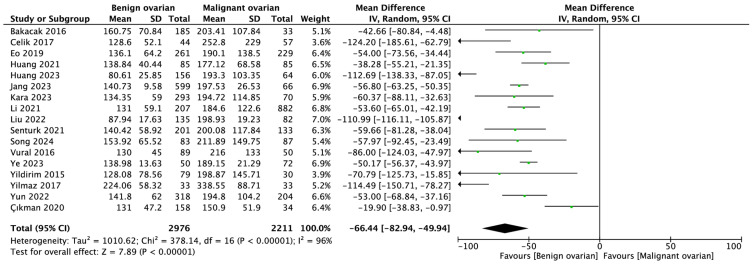
Forest plot demonstrating PLR values among benign and malignant ovarian cancer patients [26,27,28,29,30,31,32,33,34,35,37,38,39,42,44,45,46].

In the comparison of benign versus malignant ovarian cancer groups, the pooled PLR values were 134.77 ± 54.54 and 194.21 ± 120.19, respectively (MD = −66.44; 95% CI: −82.94 to −49.94; *p* < 0.001; Figure 4).

**Figure 5:**
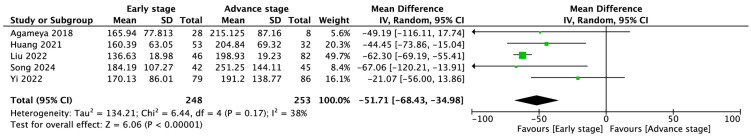
Forest plot demonstrating PLR values among patients in early and advanced stages of ovarian cancer [25,30,35,38,43].

Furthermore, the pooled PLR value in early-stage ovarian cancer was 163.74 ± 77.68, which was statistically significantly lower than in advanced-stage ovarian cancer (206.86 ± 107.24; MD = −51.71; 95% CI: −68.43 to −34.98; *p* < 0.001; Figure 5).

### 3.4. Diagnostic PLR Value

The Summary Receiver Operating Characteristic (SROC) curve for the positive likelihood ratio (PLR) in differentiating between benign and malignant ovarian cancer is presented in Figure S1. The PLR exhibited an area under the curve (AUC) of 0.635, indicating modest diagnostic accuracy. Using specificity weightings of 85%, 80%, and 75%, the optimal PLR thresholds for differentiating between benign and malignant ovarian cancer were determined to be 183 (sensitivity: 0.88; specificity: 0.82); 183 (sensitivity: 0.88; specificity: 0.82); and 191 (sensitivity: 0.49; specificity: 0.77), respectively. Notably, the identical thresholds for specificity weightings of 85% and 80% suggest that these weightings had a minimal impact on the balance between sensitivity and specificity. The threshold of 191, associated with a lower sensitivity and moderately high specificity, reflects a shift in diagnostic priorities toward reducing false positives.

The diagnostic performance of the platelet-to-lymphocyte ratio (PLR) was evaluated for differentiating ovarian cancer (OC) across various clinical contexts. For the differentiation between OC patients and healthy controls, the sensitivity was 73.85% (95% CI: 69.68–78.02), specificity was 61.30% (95% CI: 56.89–65.71), the positive likelihood ratio (PLR) was 1.94 (95% CI: 0.87–3.02), and the negative likelihood ratio (NLR) was 0.44 (95% CI: 0.20–0.69). The diagnostic odds ratio (DOR) for this comparison was 5.53 (95% CI: 3.82–7.25), with an area under the curve (AUC) of 0.664.

For distinguishing between benign and malignant ovarian lesions, the sensitivity was 68.22% (95% CI: 58.67–77.78), and the specificity was 61.10% (95% CI: 47.23–74.97). The PLR and NLR were 2.85 (95% CI: 1.45–4.25) and 0.59 (95% CI: 0.28–0.91), respectively, while the DOR was 10.94 (95% CI: 0.99–20.89), and the AUC was 0.647.

When assessing the ability of PLR to differentiate between early and advanced OC stages, the sensitivity was 77.92% (95% CI: 58.32–97.52), and the specificity was 86.94% (95% CI: 83.13–90.76). The PLR was calculated as 92.17 (95% CI: 39.63–144.71), while the NLR was 27.59 (95% CI: 0.40–54.78). The DOR for this comparison was 0.27 (95% CI: 0.20–0.35), with an AUC of 0.803 (Table 1).

The included studies used varying PLR cut-offs (range: 150–300), reflecting heterogeneity in assay methodologies. Subgroup analyses confirmed that diagnostic accuracy (AUC) remained consistent across studies regardless of cut-off thresholds (*p* = 0.12).

## 4. Discussion

The platelet-to-lymphocyte ratio (PLR) has garnered significant attention as a potential biomarker in ovarian cancer diagnosis and prognosis. Our meta-analysis delved into the diagnostic efficacy of PLR across various clinical scenarios, revealing its potential utility and inherent limitations. PLR is widely regarded as a surrogate marker of the systemic inflammatory response, providing valuable insights into the dynamic interplay between pro-inflammatory and tumor mechanisms within the host. PLR reflects the balance between platelet activation and lymphocytic response, both of which play crucial roles in cancer biology [47]. Platelets, beyond their classical role in hemostasis, are actively involved in the tumorigenic process. They contribute to the tumor microenvironment by releasing pro-inflammatory cytokines; growth factors, such as the platelet-derived growth factor (PDGF), platelet factor 4, transforming growth factor beta (TGFb), and vascular endothelial growth factor (VEGF); thrombospondin; and other mediators which serve as potent mitogens or adhesive glycoproteins for diverse cell types, including ovarian surface epithelium. These growth factors can promote angiogenesis, tumor cell proliferation, and their adherence to other cells, resulting in tumor development and metastasis [48]. Furthermore, platelets shield circulating tumor cells from immune surveillance, facilitating their survival and extravasation into distant tissues. Conversely, lymphocytes are central to the body’s immune defense against cancer, mediating cytotoxic effects on tumor cells and coordinating anti-tumor immune responses [49]. A reduction in the lymphocyte count, as reflected in an elevated PLR, may signify impaired immune surveillance and a weakened anti-tumor response. This imbalance suggests a predominance of pro-inflammatory and immunosuppressive processes, which collectively create an environment conducive to tumor progression, immune evasion, and metastasis. An elevated PLR, therefore, serves as an indirect indicator of this shift in the inflammatory-immune equilibrium, highlighting its potential utility as a biomarker for assessing cancer prognosis. In the clinical setting, a high PLR has been associated with worse outcomes in various malignancies, suggesting its role not only in reflecting disease activity but also in stratifying patients based on their inflammatory and immune status. This underscores the importance of understanding the mechanisms underlying PLR elevation and its implications for tumor biology, therapeutic decision-making, and patient management [50].

There are data in the literature supporting the association between an elevated PLR and cancer progression. For example, a study by Raungkaewmanee et al. found that ovarian cancer patients with a PLR of ≥200 had significantly shorter progression-free survival and overall survival compared to patients with a PLR of <200 [12].

Our analysis demonstrated that ovarian cancer patients exhibited a mean PLR of 181.53 ± 57.02, notably higher than the 128.89 ± 25.51 observed in healthy controls, yielding a mean difference of 46.84 (*p* < 0.001). This finding aligns with the existing literature suggesting that an elevated PLR is associated with adverse outcomes in ovarian cancer patients. For instance, a meta-analysis by Xu et al. reported that an elevated PLR was significantly correlated with poor overall survival (OS) in ovarian cancer patients [51].

When comparing benign and malignant ovarian lesions, our study found mean PLR values of 134.77 ± 54.54 and 194.21 ± 120.19, respectively, with a mean difference of −66.33 (*p* < 0.001). This suggests that higher PLR values are indicative of malignancy. Supporting this, a study by Prodromidou et al. found that both PLR and NLR values in ovarian cancer patients deviate from healthy controls and may indicate the stage of the disease [52].

Our findings indicate that advanced-stage ovarian cancer patients have significantly higher PLR values (206.86 ± 107.24) compared to those in early stages (163.74 ± 77.68), with a mean difference of −51.71 (*p* < 0.001). This observation is consistent with the meta-analysis conducted by Zhu et al., which suggested that higher PLR values were associated with poorer OS and progression-free survival (PFS) in ovarian cancer patients [53]. An elevated PLR has been linked to advanced disease stages and poor prognosis in ovarian cancer [54]. Advantages of PLR include its low cost, wide availability, and reproducibility. However, its limitations include variability due to comorbid conditions (e.g., autoimmune disorders) and lack of standardized cut-off values, which may limit its standalone prognostic utility.

Despite these associations, the diagnostic performance of PLR in differentiating between benign and malignant ovarian lesions demonstrated modest accuracy, with an area under the curve (AUC) of 0.635. This suggests that while PLR can serve as a supplementary biomarker, it may not be sufficient as a standalone diagnostic tool. While the AUC of 0.664 indicates moderate diagnostic accuracy, PLR alone may not be sufficiently robust for standalone clinical implementation. However, its cost-effectiveness and ease of calculation make it a valuable adjunct to existing biomarkers. Future research should explore the integration of PLR with other markers or imaging techniques to enhance its diagnostic utility in clinical practice. This is in line with the findings of Prodromidou et al., who reported that the diagnostic accuracy of PLR remains limited, with moderate sensitivity and specificity in detecting ovarian cancer [52].

In addition to the platelet-to-lymphocyte ratio (PLR), the neutrophil-to-lymphocyte ratio (NLR) has been extensively studied for its prognostic significance in ovarian cancer. An elevated NLR is associated with a heightened systemic inflammatory response, which can promote tumor progression and metastasis. A comprehensive meta-analysis by Zhu et al. demonstrated that higher pretreatment NLR and PLR values are significantly correlated with poorer overall survival (OS) and progression-free survival (PFS) in ovarian cancer patients [53]. This finding is supported by other studies. For instance, a meta-analysis involving 4910 patients found that the elevated pretreatment NLR was significantly related to a poor OS (HR: 1.50; 95% CI: 1.27–1.77) and PFS (HR: 1.53; 95% CI: 1.28–1.84) in ovarian cancer patients [55]. Furthermore, a study published in the Asian Pacific Journal of Cancer Prevention reported that a high NLR and PLR are associated with worse progression-free survival and 5-year overall survival in patients with ovarian cancer. These findings underscore the potential utility of NLR and PLR as accessible and cost-effective prognostic biomarkers in ovarian cancer management. However, it is important to note that some studies have reported conflicting results. In addition to PLR, other inflammatory markers, such as the neutrophil-to-lymphocyte ratio (NLR) and systemic immune-inflammation index (SII), have been explored in ovarian cancer. While NLR has shown utility in predicting prognosis, its diagnostic accuracy is comparable to PLR. SII, which incorporates platelet, neutrophil, and lymphocyte counts, may offer a more comprehensive assessment of systemic inflammation but requires further validation. A comparative analysis of these markers could provide deeper insights into the role of inflammation in ovarian cancer pathogenesis and help identify the most robust biomarker for clinical use. For example, a retrospective study on 166 epithelial ovarian cancer patients found that NLR and PLR were not significantly associated with OS or PFS [56]. Therefore, while an elevated NLR and PLR are generally associated with poorer survival outcomes in ovarian cancer patients, further large-scale, well-designed studies are warranted to establish standardized cut-off values and to validate their clinical utility in ovarian cancer management. Additionally, combining these inflammatory markers with other prognostic factors may provide a more comprehensive assessment of patient prognosis. Similarly, a study by Jiang et al. found that an elevated PLR was significantly associated with a poor OS and PFS in gynecologic cancers, including ovarian cancer [12].

The potential of the platelet-to-lymphocyte ratio (PLR) as a biomarker in ovarian cancer is evident; however, its modest diagnostic accuracy necessitates its use in conjunction with other diagnostic modalities to enhance clinical decision-making. Studies have shown that while PLR can serve as an indicator of systemic inflammation associated with tumor progression, its sensitivity and specificity are not sufficient for it to be a standalone diagnostic tool [52]. Combining PLR with other inflammatory markers, such as the neutrophil-to-lymphocyte ratio (NLR) and tumor-specific biomarkers like CA125, has been explored to improve diagnostic efficiency. Compared to traditional biomarkers like CA125 and HE4, PLR demonstrates lower diagnostic accuracy but offers the advantage of being a readily available and inexpensive marker. A combined approach, integrating PLR with CA125 or HE4, could potentially improve sensitivity and specificity, particularly in early-stage disease, where traditional biomarkers often underperform. Combining PLR with CA125 or HE4 may enhance diagnostic accuracy. For instance, Guo et al. reported a sensitivity of 85% and specificity of 78% when PLR was integrated with CA125, outperforming either marker alone [57]. Future studies should investigate the optimal combination of these markers to maximize diagnostic efficacy. For instance, a study found that the combined use of CA125, NLR, and PLR was more effective in distinguishing between benign, borderline, and malignant epithelial ovarian tumors compared to using each marker individually [33]. Further large-scale, well-designed studies are warranted to establish standardized PLR cut-off values and to validate its clinical utility in ovarian cancer management. Standardization is crucial to ensure consistency and reliability in clinical practice. Additionally, exploring the combination of PLR with other inflammatory markers and tumor-specific biomarkers may provide a more comprehensive diagnostic and prognostic tool for ovarian cancer. Integrating PLR with advanced diagnostic technologies, such as the MasSpec Pen, which offers a rapid and precise molecular analysis of tissues, could further enhance diagnostic accuracy and patient outcomes. The integration of PLR with emerging technologies, such as the MasSpec Pen, is currently a speculative suggestion, as there are no published data directly supporting this combination. However, given MasSpec Pen’s ability to provide real-time molecular profiling of tissues, future studies could explore its potential synergy with inflammatory markers like PLR to enhance diagnostic accuracy and provide a more comprehensive assessment of ovarian cancer. This meta-analysis focused on PLR as a hematologic parameter. Proteomic studies of platelet/lymphocyte activity and HLA associations, while mechanistically relevant, were excluded due to their focus on molecular pathways beyond the scope of this review.

## 5. Conclusions

These findings highlight the utility of PLR as a diagnostic marker in differentiating OC from healthy individuals, benign from malignant ovarian lesions, and early from advanced OC stages, with varying degrees of sensitivity, specificity, and diagnostic accuracy, depending on the clinical context. Notably, integrating PLR with transvaginal ultrasound or the ROMA index could improve risk stratification, especially in premenopausal women, for whom CA125 alone is less reliable.

## Figures and Tables

**Table 1:** Diagnostic performance metrics for differentiating ovarian cancer and related conditions.

Objective	Sensitivity [95%CI]	Specificity [95%CI]	PLR [95%CI]	NLR [95%CI]	DOR [95%CI]	AUC
Platelet-to-lymphocyte ratio for differentiations between OC and healthy patients	73.85% (69.68–78.02)	61.30% (56.89–65.71)	1.94 (95% CI: 0.87–3.02)	0.44 (95% CI: 0.20–0.69)	5.53 (95% CI: 3.82–7.25)	0.664
Platelet-to-lymphocyte ratio for differentiations between benign vs. malignant OC	68.22% (95% CI: 58.67–77.78)	61.10% (95% CI: 47.23–74.97)	2.85 (95% CI: 1.45–4.25)	0.59 (95% CI: 0.28–0.91)	10.94 (0.99–20.89)	0.635
Platelet-to-lymphocyte ratio for differentiations between early vs. advanced OC stage	77.92 (95% CI: 58.32–97.52)	86.94% (95% CI: 83.13–90.76)	92.17 (95% CI: 39.63–144.71)	27.59 (95% CI: 0.40–54.78)	0.27 (95% CI: 0.20–0.35)	0.803

Legend: AUC: area under the curve; CI: confidence interval; DOR: diagnostic odds ratio; NLR: negative likelihood ratio; PLR: positive likelihood ratio; OC: ovarian cancer.

## Data Availability

The data that support the findings of this study are available on request from the corresponding author (L.S.).

## Supplementary Materials

The following supporting information can be downloaded at: https://www.mdpi.com/article/10.3390/ijms26051841/s1.

## Abbreviations

AUC	Area under the curve
CBC	Complete blood count
CI	Confidence interval
CTCs	Circulating tumor cells
DOR	Diagnostic odds ratio
HR	Hazard ratio
NETs	Neutrophil extracellular traps
NLR	Neutrophil-to-lymphocyte ratio
NPV	Negative predictive value
OC	Ovarian cancer
OS	Overall survival
PFS	Progression-free survival
PLR	Platelet-to-lymphocyte ratio
PPV	Positive predictive value
SD	Standard deviation
SEN	Sensitivity
SPE	Specificity
TP53	Tumor protein p53

## References

[1] Siegel R.L., Miller K.D., Fuchs H.E., Jemal A. (2023). Cancer statistics, 2023. CA Cancer J. Clin..

[2] GLOBOCAN 2020 Database of the International Agency for Research on Cancer (IARC). https://gco.iarc.fr/en.

[3] Jemal A., Ward E.M., Johnson C.J., Cronin K.A., Ma J., Ryerson B., Mariotto A., Lake A.J., Wilson R., Sherman R.L. (2021). Annual Report to the Nation on the Status of Cancer, 1975–2021. Cancer.

[4] Pujade-Lauraine E., Banerjee S., Pignata S. (2019). Management of ovarian cancer: Present and future directions. Ann. Oncol..

[5] Maioru O.V., Radoi V.E., Coman M.C., Hotinceanu I.A., Dan A., Eftenoiu A.E., Burtavel L.M., Bohiltea L.C., Severin E.M. (2023). Developments in Genetics: Better Management of Ovarian Cancer Patients. Int. J. Mol. Sci..

[6] Cramer D.W. (2023). Incessant ovulation: A review of its importance in predicting cancer risk. Front. Oncol..

[7] Barnard M.E., Farland L.V., Yan B., Wang J., Trabert B., Doherty J.A., Meeks H.D., Madsen M., Guinto E., Collin L.J. (2024). Endometriosis Typology and Ovarian Cancer Risk. JAMA.

[8] Golara A., Kozłowski M., Cymbaluk-Płoska A. (2024). The Role of Circulating Tumor DNA in Ovarian Cancer. Cancers.

[9] Lycke M., Kristjansdottir B., Sundfeldt K. (2018). A multicenter clinical trial validating the performance of HE4, CA125, risk of ovarian malignancy algorithm and risk of malignancy index. Gynecol. Oncol..

[10] Ultimescu F., Hudita A., Popa D.E., Olinca M., Muresean H.A., Ceausu M., Stanciu D.I., Ginghina O., Galateanu B. (2024). Impact of Molecular Profiling on Therapy Management in Breast Cancer. J. Clin. Med..

[11] Chakra M.A., Lassila R., El Beayni N., Mott S.L., O’Donnell M.A. (2025). Prognostic role of the neutrophil/lymphocyte ratio in high-risk BCG-naive non-muscle-invasive bladder cancer treated with intravesical gemcitabine/docetaxel. BJU Int..

[12] Raungkaewmanee S., Tangjitgamol S., Manusirivithaya S., Srijaipracharoen S., Thavaramara T. (2012). Platelet to lymphocyte ratio as a prognostic factor for epithelial ovarian cancer. J. Gynecol. Oncol..

[13] Templeton A.J., Ace O., McNamara M.G., Al-Mubarak M., Vera-Badillo F.E., Hermanns T., Seruga B., Ocaña A., Tannock I.F., Amir E. (2014). Prognostic role of platelet to lymphocyte ratio in solid tumors: A systematic review and meta-analysis. Cancer Epidemiol. Biomark. Prev..

[14] Ohe Y., Fushida S., Yamaguchi T., Kinoshita J., Saito H., Okamoto K., Nakamura K., Tajima H., Ninomiya I., Ohta T. (2020). Peripheral Blood Platelet-Lymphocyte Ratio Is Good Predictor of Chemosensitivity and Prognosis in Gastric Cancer Patients. Cancer Manag. Res..

[15] Zhu Y., Si W., Sun Q., Qin B., Zhao W., Yang J. (2017). Platelet-lymphocyte ratio acts as an indicator of poor prognosis in patients with breast cancer. Oncotarget.

[16] Elhami A., Mobed A., Soleimany R., Yazdani Y., Kazemi E.S., Mohammadi M., Saffarfar H. (2024). Sensitive and Cost-Effective Tools in the Detection of Ovarian Cancer Biomarkers. Anal. Sci. Adv..

[17] Jiang S., Liu J., Chen X., Zheng X., Ruan J., Ye A., Zhang S., Zhang L., Kuang Z., Liu R. (2019). Platelet–lymphocyte ratio as a potential prognostic factor in gynecologic cancers: A meta-analysis. Arch. Gynecol. Obstet..

[18] Page M.J., McKenzie J.E., Bossuyt P.M., Boutron I., Hoffmann T.C., Mulrow C.D., Shamseer L., Tetzlaff J.M., Akl E.A., Brennan S.E. (2021). The PRISMA 2020 statement: An updated guideline for reporting systematic reviews. BMJ.

[19] Zhao Z., Zhao X., Lu J., Xue J., Liu P., Mao H. (2018). Prognostic roles of neutrophil to lymphocyte ratio and platelet to lymphocyte ratio in ovarian cancer: A meta-analysis of retrospective studies. Arch. Gynecol. Obstet..

[20] Stang A. (2010). Critical evaluation of the Newcastle-Ottawa scale for the assessment of the quality of nonrandomized studies in meta-analyses. Eur. J. Epidemiol..

[21] Hozo S.P., Djulbegovic B., Hozo I. (2005). Estimating the Mean and Variance from the Median, Range, and the Size of a Sample. BMC Med. Res. Methodol..

[22] Halme A.L.E., McAlpine K., Martini A. (2023). Fixed-effect versus random-effects models for meta-analyses: Random-effects models. Eur. Urol. Focus.

[23] Higgins J.P.T., Altman D.G., Gøtzsche P.C., Jüni P., Moher D., Oxman A.D., Savovic J., Schulz K.F., Weeks L., Sterne J.A.C. (2011). The Cochrane Collaboration’s Tool for Assessing Risk of Bias in Randomised Trials. BMJ.

[24] Doi S.A., Barendregt J.J., Khan S., Thalib L., Williams G.M. (2015). Advances in the meta-analysis of heterogeneous clinical trials II: The quality effects model. Contemp. Clin. Trials.

[25] Agameya A.M., Labib K., Moiet F. (2018). Using platelet-to-lymphocyte ratio as a diagnostic marker in malignant ovarian tumors. Int. J. Reprod. Contracept. Obstet. Gynecol..

[26] Bakacak M., Serin S., Ercan Ö., Köstü B., Bostancı M.S., Bakacak Z., Kıran H., Kıran G. (2016). Utility of preoperative neutrophil-to-lymphocyte and platelet-to-lymphocyte ratios to distinguish malignant from benign ovarian masses. J. Turk. Ger. Gynecol. Assoc..

[27] Celik F., Kose M. (2017). Increased neutrophil-to-lymphocyte and platelet-to-lymphocyte ratios can be used to distinguish ovarian masses. Eur. J. Gynaecol. Oncol..

[28] Çıkman M.S., Gün I., Sakin O., Koyuncu K., Anğın A.D., Karateke A., Özkaya E. (2020). Is There a Way to Predict Granulosa Cell Tumor of the Ovary? The Role of Peripheral Blood Test Parameters. Med. J. Bakirkoy.

[29] Eo W.K., Kim K.H., Park E.J., Kim H.Y., Kim H.B., Koh S.B., Namkung J. (2018). Diagnostic accuracy of inflammatory markers for distinguishing malignant and benign ovarian masses. J. Cancer.

[30] Huang H., Wu K., Chen L., Lin X. (2021). Study on the Application of Systemic Inflammation Response Index and Platelet-Lymphocyte Ratio in Ovarian Malignant Tumors. Int. J. Gen. Med..

[31] Huang K., Xu S., Wang J., Ge L., Xu J., Jia X. (2023). Combined use of CA125, neutrophil/lymphocyte ratio and platelet/lymphocyte ratio for the diagnosis of borderline and malignant epithelial ovarian tumors. J. Ovarian Res..

[32] Jang T.K., Kim H., Eo W., Kim K.H., Lee C.M., Kim M. (2023). Clinical Significance of the Combination of Serum HE4 Levels, Hemoglobin-to-Red Cell Distribution Width Ratio, and CT Imaging for the Pretreatment Assessment of Adnexal Masses. J. Cancer.

[33] Kara S.S., Keles E., Sivri Aydin D., Mat E., Yildiz G., Temocin R.B., Birol Ilter P., Yildiz P. (2023). Utility of preoperative neutrophil–lymphocyte and platelet–lymphocyte ratios in differential diagnosis of benign, borderline, and malignant ovarian tumors. J. Exp. Clin. Med..

[34] Li L., Tian J., Zhang L., Liu L., Sheng C., Huang Y., Zheng H., Song F., Chen K. (2021). Utility of Preoperative Inflammatory Markers to Distinguish Epithelial Ovarian Cancer from Benign Ovarian Masses. J. Cancer.

[35] Liu Z., Wu J., Wang X., Ji X. (2022). Multivariate logistic regression analysis of the correlation between ve biomarkers and ovarian cancer in patients with intermediate-risk: A prospective cross-sectional study. Front. Cell Dev. Biol..

[36] Ozaksit G., Tokmak A., Kalkan H., Yesilyurt H. (2015). Value of the platelet to lymphocyte ratio in the diagnosis of ovarian neoplasms in adolescents. Asian Pac. J. Cancer Prev..

[37] Senturk M., Oge T., Yalcin O.T. (2021). Mean Platelet Volume, Neutrophil to Lymphocyte Ratio and Platelet to Lymphocyte Ratio for Differentiating Benign, Borderline and Malignant Ovarian Masses. Sabuncuoglu Serefeddin Health Sci..

[38] Song L., Wu Q., Bai S., Zhao J., Qi J., Zhang J. (2024). Comparison of the diagnostic efficacy of systemic inflammatory indicators in the early diagnosis of ovarian cancer. Front. Oncol..

[39] Vural F., Aka N., Ertas S., Kose G., Tufekci E.C. (2016). The ovarian cancers in geriatric population: The validity of inflammatory markers, malignancy risk indices 1, 2, 3, 4, and CA-125 levels in malignancy discrimination of adnexal masses. Eur. J. Gynaecol. Oncol..

[40] Wang N., Li C., Yang Y., Guan Y., Wang F., Wang Y., Zhao W. (2021). The Use of Platelet/Lymphocyte Ratio and Cancer Antigen. 125 Combined with Magnetic Resonance Diffusion-Weighted Imaging in Diagnosis of Recurrent Ovarian Cancer and Neuropathic Pain. World Neurosurg..

[41] Wang M., Lv X., Wang Y., Li Y., Li H., Shen Z., Zhao L. (2024). Biomarkers of peripheral blood neutrophil extracellular traps in the diagnosis and progression of malignant tumors. Cancer Med..

[42] Ye L., Zhou G., Zhou L., Wang D., Xiong S., Liu C., Zhang G. (2023). Diagnostic roles of neutrophil-to-lymphocyte ratio, monocyte-to-lymphocyte ratio, platelet-to-lymphocyte ratio, C-reactive protein, and cancer antigen 125 for ovarian cancer. J. Int. Med. Res..

[43] Yi Q., Ran Y., Li C. (2022). Diagnostic value of serum tumor markers for epithelial ovarian cancer stage I-II—A retrospective analysis. Authorea.

[44] Yildirim M., Demir Cendek B., Filiz Avsar A. (2015). Differentiation between benign and malignant ovarian masses in the preoperative period using neutrophil-to-lymphocyte and platelet-to-lymphocyte ratios. Mol. Clin. Oncol..

[45] Yilmaz E., Coskun E.I., Sahin N., Ciplak B., Ekici K. (2017). MPV, NLR, and platelet count: New hematologic markers in diagnosis of malignant ovarian tumor. Eur. J. Gynaecol. Oncol..

[46] Yun T.H., Jeong Y.Y., Lee S.J., Choi Y.S., Ryu J.M. (2022). Neutrophil–Lymphocyte and Platelet–Lymphocyte Ratios in Preoperative Differential Diagnosis of Benign, Borderline, and Malignant Ovarian Tumors. J. Clin. Med..

[47] Dan J., Tan J., Huang J., Yuan Z., Guo Y. (2023). Early changes of platelet lymphocyte ratio correlate with neoadjuvant chemotherapy response and predict pathological complete response in breast cancer. Mol. Clin. Oncol..

[48] Liao K., Zhang X., Liu J., Teng F., He Y., Cheng J., Yang Q., Zhang W., Xie Y., Guo D. (2023). The role of platelets in the regulation of tumor growth and metastasis: The mechanisms and targeted therapy. MedComm.

[49] Mukherjee A.G., Wanjari U.R., Namachivayam A., Murali R., Prabakaran D.S., Ganesan R., Renu K., Dey A., Vellingiri B., Ramanathan G. (2022). Role of Immune Cells and Receptors in Cancer Treatment: An Immunotherapeutic Approach. Vaccines.

[50] Rychlik U., Szatkowski W., Tarapacz J., Wójcik E., Stasik Z., Brandys K., Kulpa J. (2017). Wartość predykcyjna wskaźników stanu zapalnego (NLR, LMR i PLR) u chorych na raka jajnika przed leczeniem I-rzutu. Diagn. Lab..

[51] Xu X., Wang W., Yang M., Song L., Xiong J., Lin J., Long J., Bai Y., Zheng Y., Zhao L. (2018). Prognostic significance of the platelet-to-lymphocyte ratio in ovarian cancer: A meta-analysis. Transl. Cancer Res..

[52] Prodromidou A., Andreakos P., Kazakos C., Vlachos D.E., Perrea D., Pergialiotis V. (2017). The diagnostic efficacy of platelet-to-lymphocyte ratio and neutrophil-to-lymphocyte ratio in ovarian cancer. Inflamm. Res..

[53] Zhu Y., Zhou S., Liu Y., Zhai L., Sun X. (2018). Prognostic value of systemic inflammatory markers in ovarian Cancer: A PRISMA-compliant meta-analysis and systematic review. BMC Cancer.

[54] Tian C., Song W., Tian X., Sun Y. (2018). Prognostic significance of platelet-to-lymphocyte ratio in patients with ovarian cancer: A meta-analysis. Eur. J. Clin. Investig..

[55] Zhou Q., Hong L., Zuo M.Z., He Z. (2017). Prognostic significance of neutrophil to lymphocyte ratio in ovarian cancer: Evidence from 4,910 patients. Oncotarget.

[56] Winata I.G.S., Pradnyana I.W.A.S., Yusrika M.U., Pradnyaandara I.G.B.M.A., Pradnyadevi P.A.S. (2024). Neutrophil to Lymphocyte Ratio and Platelet to Lymphocyte Ratio as an Early Prognostic Marker in Patients with Ovarian Cancer: A Systematic Review and Meta-Analysis. Asian Pac. J. Cancer Prev..

[57] Guo C., Zhang C. (2022). Platelet-to-Lymphocyte Ratio and CA125 Level as a Combined Biomarker for Diagnosing Endometriosis and Predicting Pelvic Adhesion Severity. Front. Oncol..

